# The metaxenia effects of different pollen grains on secondary metabolites enzymes and sugars of ‘Piarom’ date palm fruit

**DOI:** 10.1038/s41598-022-14373-w

**Published:** 2022-06-16

**Authors:** Ali Reza Shahsavar, Asma Shahhosseini

**Affiliations:** Department of Horticultural Science, College of Agriculture, Shiraz University, Shiraz, Iran

**Keywords:** Biochemistry, Physiology, Plant sciences

## Abstract

In this research, the characteristics of pollen were studied in eight pollinating cultivars of date palm, namely, ‘Shahani’, ‘Kabkab’, ‘Zahedi’, ‘Beraem’, ‘Faryab’, ‘Sheikhali’, ‘Fard’ and ‘Jarvis’. The characteristics were compared and metaxenia effects were evaluated on secondary metabolites, enzymes and other biochemical compounds of ‘Piarom’ date fruits. The evaluations were carried out during four stages of fruit growth and development. The pollen of these eight pollinating cultivars were compared in terms of carbohydrates, proteins, starch, total phenol, flavonoids, pectin methyl esterase, and amylase enzymes. According to the results, the pollen of ‘Sheikhali’, ‘Fard’, ‘Zahedi’ and ‘Shahani’ cultivars contained more of the above compounds, compared to the other cultivars. Regarding the effects of pollen on the composition of ‘Piarom’ date fruits, ‘Fard’ and ‘Sheikhali’ pollen produced the lowest amount of soluble tannin, which resulted in a better quality of ‘Piarom’ date fruits. Pollen was also obtained from ‘Sheikhali’ and ‘Fard’ cultivars for evaluations, showing that they led to the highest amounts of glucose and fructose in the fruits. Regarding the sucrose amount, ‘Jarvis’ and ‘Shikhali’ produced the best results. Pollen of ‘Sheikhali’ and ‘Fard’ cultivars caused the lowest amount of chlorophyll at the different stages of fruit growth, indicating a better decomposition of fruit chlorophyll and, as a result, better fruit quality. Pollen of ‘Sheikhali’ and ‘Fard’ cultivars produced the highest amounts of secondary metabolites such as total phenol, carotenoids and anthocyanin at the different stages of fruit development. The pollen of ‘Fard’ and ‘Sheikhali’ cultivars produced the highest levels of polygalacturonase, cellulase and invertase enzymes at different growth stages of the ‘Piarom’ date fruit. Regarding cellulase enzyme, fruits of the ‘Zahedi’ cultivar had more cellulase than the fruits of ‘Sheikhali’. In general, the pollen of ‘Fard’ and ‘Sheikhali, in comparison with other cultivars, improved the quantity and quality of ‘Piarom’ date fruits, due to their metaxenia properties.

## Introduction

Date palm (*Phoenix dactylifera* L.) is a monocotyledonous plant, belonging to the Palmaceae family, and is a dioecious species, with separate female and male trees. The date flowers are unisexual. Pistillate and staminate flowers are born in a big inflorescence called spadix or spike. The inflorescence in its early stages is enclosed in a hard covering known as a spathe^1^. Male date palm flowers are white and usually have three sepals, three petals and six stamens. Female flowers are usually yellowish green and have three sepals, three petals, six staminodes and three separate carpels, although only one of them develops into a fruit. When the temperature reaches over 18 °C, the date palm trees begin to flower. The male palms normally bloom from February to March, and the female trees do slightly later in March and April. In date palm, spathe cracking is equivalent to anthesis, and this is when the flowers are ready for pollination and fertilization. When anthesis, the inflorescence push to the spathe wall, causing the spathe to crack longitudinally so that the flowers appear^2^. After spathe cracking, the stamens of male flowers release the pollen grains and the stigma of female flowers become receptive.

Date palm pollination by humans can play a fundamental role in the quantity and quality of products. Pollen of different date cultivars are very different in shape, size, weight, germination percentage and their constituents^3^. Due to their metaxenia properties, they have significant effects on fruit characteristics of the same year^4^. Therefore, in pollination of dates, by selecting the appropriate pollen, important traits of the fruit, including its constituent compounds, can be influenced.

Date fruit is a source of secondary metabolites, such as anthocyanins, phenols, carotenoids, and flavonoids, which have protective roles against oxidative stress^5^. A research on antioxidant properties of date fruits showed that phenolic compounds, including phenolic acids and flavonoids, mainly prevent oxidative damage to lipids, nucleic acids, and proteins by inhibiting free radicals^6^. The antioxidant activity of date fruits is attributed to the presence of a large amount of phenolic compounds^7^. Phenolic compounds are reduced during the developmental stages of date fruit, reaching a minimum during the stages of maturity and ripening of the fruit^8^.

Date fruits undergo significant physical and chemical changes after formation, until they reach the final stage of growth. These changes include four stages, which are based on Arabic terms, i.e. kimri, khalal, rutab, and tamar. In the kimri stage, the fruit color is green, the weight and volume of the fruit rapidly increase, the taste of the fruit is astringent, the fruit juice increases and, at the end of this stage, the fruit reaches its maximum growth. The khalal stage is accompanied by a change in the color of the fruit, which changes from green to beige, yellow, and red. This is when the juice and weight of the fruit decrease. In the rutab stage, the color of the fruit darkens and texture softens. Astringency decreases and the amount of sugar increases. The fruit ripens and becomes edible. In the tamar stage, the fruit has the least amount of water and the highest amount of sugar. At this stage, the vital activities of the fruit are stopped^9,10^.

Based on a study on dates, it was revealed that the khalal stage has the highest amount of phenolic, flavonoid, and tannin substances as well as antioxidant activity compared to the two stages of rutab and tamar^11^. According to a study by Besbes et al.^12^, although the difference in the amount of total phenol in the fruits, of different date cultivars, is influenced by factors such as cultivar, growth conditions, and geographical conditions, using pollen grains in pollination operations can play an important role. Chloroplasts of all higher plants have important biomarkers, including chlorophyll a, chlorophyll b, and carotenoids, which are able to absorb effective light energy during photosynthesis^13^. Carotenoids, such as anthocyanins, polyphenols, and flavonoids, are low molecular weight antioxidants that play an effective role in neutralizing free radicals; that is, they protect intracellular compounds against the damaging effects of free radicals^14^. Chlorophyll a and carotenoids are more susceptible to stress conditions than chlorophyll b^15^. Anthocyanins are the largest group of water-soluble pigments after chlorophyll, and are naturally found in many plants^16^. They are an important part of plant secondary metabolites and chemically belong to the group of flavonoids with antioxidant properties^17^. Date fruits contain a variety of compounds, including sugars, hormones, enzymes, secondary metabolites, and antioxidant compounds that undergo many changes during different stages of date development from kimri to tamar. Considering the effect of metaxenia on date fruits and the effect of pollen characteristics on fruit composition in the same year, there was a need for more in-depth research on the relationship between pollen type and fruit composition. The ‘Piarom’ date is a semi-dry, late ripening cultivar with long, elliptical fruits that are approximately 3 to 5 cm long, brown in color and have thin skin. This cultivar is known worldwide because of its high quality, and it is nationally a famous cultivar in date orchards of Iran. If the quality of the fruit improves, it can have a significant impact on global markets. It has the highest price among the different cultivars of dates.

The aim of this study was to investigate the pollen grain compositions of eight pollinating cultivars and their metaxenia relationship with secondary metabolites, enzymes and other biochemical compounds of ‘Piarom’ date fruit at different stages of growth and development.

## Materials and methods

This research was conducted in a well-known date palm orchard in Jahrom city, Fars province, Iran, for two consecutive years (2016–2017). In this experiment, pollen of eight pollinating cultivars were used, namely, ‘Sheikhali’, ‘Faryab’, ‘Beraem’, ‘Zahedi’, ‘Shahani’ and ‘Kabkab’ cultivars. Meanwhile, ‘Jarvis’ and ‘Fard’ cultivars were regarded as international pollinators. Their pollen were used for pollinating 32 twelve-year-old ‘Piarom’ date trees. There were four replications; in each replication, there was one tree. Pollen grains were obtained during ripening of male spathes in March from male date palm trees that grew in the Saadabad Date Palm Research Station located in Saadabad city of Bushehr province. Male spathes were collected with permission from the Agricultural and Natural Resources Research Center of Bushehr Province. The spathes of each cultivar were separated from male trees and transferred to the orchard of the ‘Piarom’ date palm trees for pollination. To prevent unwanted pollination, ‘Piarom’ date inflorescences were covered with kraft paper before pollination. During pollination, three strands of male inflorescences were placed inside each female inflorescence and covered again with a paper bag for 4 weeks. The use of plants and plant organs in this research complied with international, national and institutional guidelines.

### Measurement of pollen grain composition

#### Carbohydrate and starch

To measure the amount of carbohydrates in pollen of the different cultivars, 0.1 g of pollen was homogenized in 80% ethanol. The supernatant was then dried in an oven at 40 °C and dissolved by adding water. The amount of soluble sugars was determined by injecting it into reverse-phase high-performance liquid chromatography (HPLC) (Portuguese Unicam model) with a Fast Carbohydrate column (Bio-Rad, Hercules, CA, USA) which operated at 85 °C, with de-ionized, degassed water as an eluent mobile phase^18^.

To measure the amount of starch, 2 mL of hydrochloric acid was added to the remaining solution from the ethanol extract, was prepared for measuring carbohydrates and boiled for 30 min. After cooling, the pH was reduced to 4.5 by adding potassium hydroxide to the solution. To enable the digestion of starch, 36 units of amyloglucosidase were added to the solution and were stored at 50 °C for 60 min. Finally, the solution was kept at 25 °C for 30 min, and the absorption rate was read at 340 nm^19^.

#### Protein

To measure the protein, 0.05 g of pollen were homogenized with 2 mL of 0.1 M phosphate buffer with an acidity of 6.8 and centrifuged for 15 min. Then, 50 μL of the supernatant was removed and the volume was increased to 100 µL. Finally, 5 mL of Bradford solution was added, and the absorbance of the solution was read at 595 nm by using a spectrophotometer^20^.

#### Total phenol

The amount of total phenol in the pollen of different cultivars was measured by preparing an ethanol (96%) extract of pollen grains and using the Folin-Ciocalteu reagent colorimetric method. In this method, granulated ethanol extract (0.5 mL) was mixed with 2.5 mL of Folin-Ciocalteu reagent (diluted 1: 10 with distilled water) and 2 mL of 4% sodium carbonate. The solution was placed in the dark for 2 h and then the absorbance was measured at 740 nm. The amount of total phenol was expressed in terms of mg of gallic acid per gram of plant dry weight^21^.

#### Flavonoids

To measure flavonoids in the pollen of different cultivars, 0.5 mL of ethanol extract (4.3 mol of 80% ethanol) was mixed with one tenth of a milliliter of aluminum nitrate and 0.1 mL of 1M potassium acetate. After 40 min, the absorbance was measured at 430 nm using a spectrophotometer and the amount of total flavonoids was expressed in mg of quercetin per gram of dry weight of plant matter^22^.

#### Pectin methylesterase (PME)

To measure the activity of PME enzyme in the pollen grains of different cultivars, 50 mg of pollen were added to 250 μL of PME extraction buffer containing 0.1 M citrate and 0.2 M sodium phosphate buffer and 0.1 M sodium chloride (pH 5), which were centrifuged for 10 minutes at 14,000 rpm. The supernatant was collected and one mL of the extract was removed, to which 0.2 M sodium phosphate was added (pH 6.3) and incubated at 37 °C for 24 h. Then, 0.2 mL of 0.05% red ruthenium and 0.5 mL of 0.6 M calcium chloride were added to the solution and centrifuged for 15 minutes at 14,000 rpm. Finally, the supernatant was collected, and the absorption rate was measured at 534 nm by using a spectrophotometer^23^.

#### Amylase

To measure the activity of amylase enzyme in the pollen of different cultivars, pollen grains (1 g) were homogenized with 15 mL of sodium citrate buffer at pH 5. The solution was then centrifuged at 10,000 rpm for 10 min. The 0.5 mL of the resulting extract was added to 3.5 mL of hydrochloric acid solution, and the absorbance was read at 580 nm by using a spectrophotometer^24^.

### Measurement of ‘Piarom’ date fruit compositions

#### Fruit chlorophyll and carotenoid

To measure the amount of chlorophyll and carotenoids in the fruit at four stages of development (kimri, khalal, rutab, and tamar), 1 g of the outer layer of fruit flesh was removed and extracted with 80% acetone and then centrifuged for 10 min. The absorbance of the supernatant at 663 nm (chlorophyll a), 646 nm (chlorophyll b), and 470 nm (carotenoid) was measured using a spectrophotometer. Finally, using the following formulas, the amounts of chlorophyll and carotenoids were calculated in terms of mg/g wet weight of the sample^25^.

$$\begin{gathered} {\text{Chlorophyll a }} = \, \left( {{19}.{3 } \times {\text{ A663 }}{-} \, 0.{86 } \times {\text{ A646}}} \right){\text{ V}}/{\text{W}} \hfill \\ {\text{Chlorophyll b }} = \, \left( {{19}.{3 } \times {\text{ A646 }}{-}{ 3}.{6 } \times {\text{ A663}}} \right){\text{ V}}/{\text{W}} \hfill \\ {\text{Carotenoids }} = { 1}00\left( {{\text{A47}}0} \right) \, {-}{ 3}.{27}\left( {{\text{mg chl}}.{\text{ a}}} \right) \, {-}{ 1}0{4}\left( {{\text{mg chl}}.{\text{ b}}} \right)/{227} \hfill \\ \end{gathered}$$

V = Volume of supernatant; A = Light absorption at 663, 646, and 470 nm; W = Wet weight of sample in term of gr.

#### Fruit reducing and non-reducing sugars

To measure reducing and non-reducing sugars (glucose, fructose, and sucrose), date fruit flesh texture in tamar stage was divided into small pieces and homogenized with 100 mL of distilled water and kept at room temperature for 1 h. The resulting extract was centrifuged at 10,000 rpm for 15 min. Then, 500 μL of the supernatant was removed and diluted with 500 μL of distilled water and was increased to 4 mL with 3 mL of acetonitrile. The prepared solution was passed through a 0.2 μM membrane filter. Glucose, fructose, and sucrose standards were also prepared at concentrations of 2.5, 5, 10, 15, and 20 mg/L. Then, 25 μL of the standards and extracts of date fruit were injected into the HPLC (Portuguese Unicam model) with a C18 column and de-ionized water mobile phase. Finally, the glucose, fructose, and sucrose levels were calculated^26^.

#### Fruit soluble tannin

To measure the soluble tannins, 5 g of fruit skin and flesh tissue were sampled in the four stages of development (kimri, khalal, rutab, and tamar) and were homogenized with 25 mL of 80% methanol in a porcelain mortar. Then, they were centrifuged at 360 rpm for 5 min. The supernatant was separated, and the precipitate was extracted with 80% methanol and centrifuged. The supernatants were added together and made up to 100 mL with deionized water. One milliliter of the resulting solution was mixed with 6 mL of deionized water and half a milliliter of Folin-Ciocalteu reagent (previously diluted 1:10 with distilled water). After 3 min, 1 mL of saturated sodium carbonate and 1.5 mL of deionized water was added and, after 1 h, the amount of light absorption of the solution at 750 nm was read using a spectrophotometer. The concentration of soluble tannin was calculated based on the standard curve of different concentrations of pure gallic acid which was prepared at the same time as the samples^27^.

#### Fruit total phenol

To measure total phenol, 200 mg of fruit skin and flesh tissue were sampled in the four stages of development (kimri, khalal, rutab, and tamar) and were mixed with 2 mL of 50% ethanol in a porcelain mortar. After storage for 1 h, the resulting solution was centrifuged for 10 min. Then, 200 µL of the solution was mixed with 1.5 mL of Folin-Ciocalteu reagent (diluted 1: 10 with distilled water) and kept under the same conditions for 5 min, followed by the addition of 20% sodium carbonate (1.5 mL). After 90 min, the absorbance of the solution was measured at 750 nm using a spectrophotometer. The concentration of total phenol was calculated based on a standard curve of different concentrations of pure gallic acid^28^.

#### Fruit anthocyanin

To measure anthocyanin, 0.5 g of fruit peel was sampled in the four stages of fruit development (kimri, khalal, rutab, and tamar) and were homogenized with 25 mL ethanol hydrochloric acid solution (980 mL 95% ethanol + 20 mL concentrated hydrochloric acid). Finally, the optical density of the solution was measured at 530 nm using a spectrophotometer. The amount of anthocyanin was measured in mg per 100 g of fruit peel according to the following equation^29^.

$${\text{Anthocyanin in 1}}00{\text{ g of fruit peel }}\left( {{\text{mg}}} \right) = \frac{{{\text{optical density }} \times {\text{ volume of extracted solution }} \times { }100}}{{{\text{weight of the sample }} \times { }98.2}}$$

#### Fruit cellulase enzyme

To measure cellulase activity, 50 g of fruit mesocarp tissue were sampled in the four stages of fruit development (kimri, khalal, rutab, and tamar) and were mixed in 85 mL of 12% polyethylene glycol and 2% sodium bisulfite for one minute. After centrifugation for 10 min at 10,000 rpm, the resulting material was divided into two parts to measure the enzyme activity. The resulting material was mixed with 50 mM sodium acetate (pH 5) and with 0.5 M sodium chloride at 4 °C in a shaker for 1 h. After being centrifuged, the supernatant was separated and diluted by 5 mM sodium acetate at pH 5. By measuring the amount of reductant groups isolated from carboxymethylcellulose, the degree of cellulase activity was determined. The reaction mixture contained 0.25 mL of pure enzyme, 0.5 mL of 1% carboxymethylcellulose, 0.25 mL of 100 mM sodium acetate buffer (pH 5) and was incubated at 37 °C for 1, 2, 3, 4, and 5 h. The precursor was added to the control tubes after heat treatment, and the samples were incubated in a hot water bath for 5 min, the amount of absorption was measured at 550 nm. For determining the concentrations of reducing groups, the D-glucose standard was used. Cellulase activity as a unit of enzymatic activity was defined as the amount of enzyme isolated under standard conditions from one micromole of glucose per hour^30^.

#### Fruit invertase enzyme

To measure the amount of invertase enzyme in fruits at each of the four stages of growth and development (kimri, khalal, rutab, and tamar), 5 g of fruit samples were homogenized in a mortar using sodium acetate extraction buffer. After two centrifuges, a dinitrosalicylic acid solution was added to form a color solution. Color samples were recorded using a spectrophotometer at 540 nm, and the data was expressed in micromoles per gram of fresh material. The extraction of invertase enzyme was similar to that of cellulase. The assay mixture consisted of 1.5 M sucrose, 1 mL enzyme extract and 0.5 M acetate buffer (pH 4.5) in a total volume of 5 mL. This mixture was incubated at 37 °C for 1 h and, accordingly, samples (1 mL) were taken at 10 min intervals during this period. The amount of reducing sugars was determined using dinitrosalicylic acid. The amount of reducing sugars was calculated after isolation, using a calibration chart, and one unit of invertase enzyme was defined as the amount of enzyme required for the hydrolysis of sucrose (0.5 μM) per minute^31^.

#### Fruit polygalacturonase enzyme

To measure the polygalacturonase enzyme, fruit tissue (2.5 g) was homogenized at each of the four stages of fruit development (kimri, khalal, rutab, and tamar) in an acidic environment (pH 3). The mixture was centrifuged for 15 min at 8000 rpm, and the supernatant was removed. The precipitate was washed with distilled water, and after centrifugation, the supernatant was removed. The resulting solution was mixed with 1.2 mol of sodium chloride in a ratio of 1:1 until its pH value reached 6. The supernatant was removed after centrifugation for 20 min at 10,000 rpm. Then, 50 μL of the extract was added to 350 μL of the buffer containing 0.2% polygalacturonic acid and 0.4 mol of sodium acetate (pH 4.4). After 10 min of incubation at 37 °C, the reaction was stopped with the buffer at a concentration of 0.1 mol and the pH equaled 9. The amount of light absorption was measured at 276 nm, and the enzyme activity in the samples was evaluated using a standard curve^32^.

### Statistical analysis

This study was performed on a total of 32 twelve-year-old trees. There were eight treatments, four replications and one tree per replicate. A randomized complete block design was used for analyzing the data. Duncan’s multiple range test was used for determining the statistical significance of the means (p < 0.05). SPSS software was used for the data analysis.

### Statement on guidelines

All experimental procedures on date palm plants complied with relevant institutional, national, and international guidelines and legislations.

## Results

In this study, the results of the first and second years were not statistically different, so the average results of the two years were reported.

### Pollen grain compositions

The highest amount of carbohydrates was obtained in the pollen of ‘Fard’, ‘Shahani’, ‘Zahedi’ and ‘Sheikhali’ cultivars which were not significantly different from the amount of carbohydrates in the pollen of ‘Jarvis’ cultivar. The lowest amount of carbohydrates was obtained in the pollen of ‘Beraem’ cultivar which was not significantly different from the amount of carbohydrates in the pollen of ‘Kabkab’ cultivar (Table 1).

**Table 1:** The pollen grain ingredients of eight pollinator cultivars of date palm.

Cultivars	Carbohydrate (%)	Protein (%)	Starch (%)	Total phenol (mg GAE/g)	Flavonoids (mg QE/g)	Pectin methyl esterase (IU/g. FW)	Amylase (IU/mg. FW)
‘Shahani’	13.33 a	30.22 ab	9.01 ab	36.11 c	9.86 a	1.41 c	80.83 b
‘Kabkab’	11.80b c	29.47 b	8.33 de	17.40 f	5.46 d	0.61 e	62.86 d
‘Zahedi’	13.19 a	31.60 a	8.83 bc	45.83 b	9.60 a	1.27 c	84.40 a
‘Beraem’	11.63 c	30.13 ab	8.20 e	19.61 ef	4.18 e	0.54 e	66.53 c
‘Faryab’	12.61 abc	29.79 ab	8.50 de	22.19 e	6.06 d	0.85 d	61.16 d
‘Sheikhali’	13.04 a	31.10 ab	8.82 bc	50.77 a	8.84 b	1.94 ab	81.40 ab
‘Fard’	13.86 a	30.74 ab	9.29 a	49.68 a	9.29 ab	1.76 b	80.80 b
‘Jarvis’	12.90 ab	30.86 ab	8.60 cd	28.40 d	8.07 c	2.10 a	67.73 c

Different letters within columns indicate significant differences according to Duncan’s multiple range test (p < 0.05).

The highest amount of starch was obtained in the pollen of ‘Fard’ cultivar which was not significantly different from the amount of starch in the pollen of ‘Shahani’ cultivar. The lowest amount of starch was obtained in the pollen of ‘Beraem’ cultivar which did not show a significant difference compared to the amount of starch in the pollen of ‘Kabkab’ and ‘Faryab’ cultivars (Table 1).

The highest amount of protein was obtained in the pollen of ‘Zahedi’ cultivar which was not significantly different from the amount of protein in the pollen of ‘Sheikhali’, ‘Jarvis’, ‘Fard’ and ‘Shahani’ cultivars. The lowest amount of protein was obtained in the pollen of ‘Kabkab’ cultivar (Table 1).

The highest amount of total phenol was obtained in the pollen of ‘Sheikhali’ and ‘Fard’ cultivars, whereas the lowest amount was obtained in the pollen of ‘Kabkab’ cultivar which, compared to other cultivars, showed a significant difference at the probability level of 5% (Table 1).

The highest amount of flavonoids was obtained in the pollen of ‘Shahani’ and ‘Zahedi’ cultivars which did not differ significantly from the amount of flavonoids in the pollen of ‘Fard’ cultivar, whereas the lowest amount was obtained in the pollen of ‘Beraem’ cultivar which, compared to other cultivars, had a significant difference at the probability level of 5% (Table 1).

The highest amount of pectin methylesterase enzyme was obtained in the pollen of ‘Jarvis’ cultivar which was not significantly different from the amount of this enzyme in the pollen of ‘Sheikhali’ cultivar. The lowest amount of pectin methylesterase was obtained in the pollen of ‘Beraem’ and ‘Kabkab’ cultivars which, compared to other cultivars, had a significant difference at the 5% probability level (Table 1).

The highest amount of amylase enzyme activity was obtained in the pollen of ‘Zahedi’ cultivar which was not significantly different from the amount of this enzyme in the pollen of ‘Sheikhali’ cultivar. In contrast, the lowest amount was obtained in the pollen of ‘Faryab’ and ‘Kabkab’ cultivars, which was significantly different from the other cultivars at a probability level of 5% (Table 1).

### Fruit compositions

#### Fruit total phenol

The highest amount of total phenol of ‘Piarom’ date fruit at the kimri stage was obtained by the pollen of ‘Jarvis’ and ‘Shahani’ cultivars, whereas the lowest amount was obtained by the pollen of ‘Kabkab’ cultivar which had a significant difference at the 5% probability level compared to the other cultivars (Table 2). During the khalal stage, the highest amount of total phenol was observed in fruits created by the pollen of ‘Jarvis’, ‘Shahani’ and ‘Sheikhali’ cultivars, whereas the lowest amount was observed in fruits created by the pollen of ‘Kabkab’ and ‘Beraem’, which was significantly different from other cultivars at a probability level of 5%. Also, in the rutab stage, the highest amount of total fruit phenol occurred from the pollen of ‘Jarvis’ and ‘Shahani’ cultivars, whereas the lowest amount was created by the pollen of ‘Kabkab’ and ‘Beraem’. In the tamar stage, the highest amount of total phenol was produced by the pollen of ‘Jarvis’ and ‘Shahani’ cultivars, whereas the lowest amount was produced by the pollen of ‘Kabkab’ and ‘Beraem’ which had a significant difference at the 5% probability level compared to ‘Shahani’ and ‘Jarvis’ cultivars. Therefore, all pollinating cultivars caused the amount of phenol of ‘Piarom’ date fruit to decrease significantly from the kimri to the tamar stage (Table 2).

**Table 2:** Total phenol content of ‘Piarom’ date fruit in kimri, khalal, rutab and tamar stages after pollination with eight pollinating cultivars.

Cultivars	Kimri (mg/100 g)	Khalal (mg/100 g)	Rutab (mg/100 g)	Tamar (mg/100 g)
‘Shahani’	152.82 a	91.74 a	43.90 a	22.47 a
‘Kabkab’	149.53 d	89.28 c	40.20 d	20.47 b
‘Zahedi’	151.91 b	90.88 b	43.04 bc	21.72 ab
‘Beraem’	150.44 c	89.42 c	40.47 d	20.50 b
‘Faryab’	151.45 b	90.41 b	42.56 c	21.24 ab
‘Sheikhali’	151.98 b	91.70 a	43.60 ab	21.94 ab
‘Fard’	151.78 b	90.75 b	42.84 bc	21.53 ab
‘Jarvis’	152.89 a	91.92 a	44.03 a	22.63 a

Different letters within columns indicate significant differences according to Duncan’s multiple range test (p < 0.05).

#### Fruit soluble tannin

The highest amount of tannin in ‘Piarom’ date fruit was produced in the kimri stage, as a result of pollination with the pollen of ‘Jarvis’ and ‘Kabkab’ cultivars, which had no significant difference in the amount of fruit tannin, compared to fruits which resulted from the pollen of ‘Beraem’ and ‘Faryab’ cultivars at the 5% probability level (Table 3). Also, the lowest amount was observed at this stage with the pollen of ‘Fard’, ‘Sheikhali’ and ‘Zahedi’ cultivars. During the khalal stage, the highest amount of fruit tannin was obtained with the pollen of ‘Kabkab’, ‘Beraem’ and ‘Faryab’ cultivars, which did not differ significantly from the amount of fruit tannin that occurred from the effect of ‘Jarvis’ pollen (at the 5% probability level). At this stage, the lowest amount of tannin was observed in fruits which resulted from the pollen of ‘Fard’ cultivar. At the rutab stage, the highest amount of tannin was observed in fruits that were created by pollen from ‘Kabkab’, ‘Beraem’, ‘Faryab’ and ‘Jarvis’ cultivars, whereas the lowest amount was observed from the effect of pollen that was sourced from ‘Fard’, ‘Sheikhali’, ‘Zahedi’ and ‘Shahani’ cultivars, which had a significant difference with other cultivars at the probability level of 5%. At the tamar stage, the highest amount of tannin in the fruits resulted from pollen that was sourced from ‘Kabkab’ and ‘Beraem’ cultivars, whereas the lowest amount was obtained with the pollen of ‘Fard’, ‘Sheikhali’ and ‘Zahedi’ which had significant differences with other cultivars at the probability level of 5%.

**Table 3:** The amount of soluble tannin in the fruit of ‘Piarom’ dates in the kimri, khalal, rutab and tamar stages after pollination with eight pollinating cultivars.

Cultivars	Kimri (mg/g)	Khalal (mg/g)	Rutab (mg/g)	Tamar (mg/g)
‘Shahani’	11.66 bc	10.76 bc	3.54 b	3.55 b
‘Kabkab’	12.17 a	11.42 a	4.22 a	4.13 a
‘Zahedi’	11.62 c	10.72 bc	3.44 b	2.86 c
‘Beraem’	12.06 ab	11.40 a	4.18 a	4.12 a
‘Faryab’	12.04 ab	11.28 a	4.09 a	3.61 b
‘Sheikhali’	11.47 c	10.65 bc	3.4 b	2.84 c
‘Fard’	11.39 c	10.59 c	3.35 b	2.75 c
‘Jarvis’	12.20 a	11.09 ab	4.06 a	3.59 b

Different letters within columns indicate significant differences according to Duncan’s multiple range test (p < 0.05).

#### Fruit chlorophyll a and b

The highest chlorophyll a content of ‘Piarom’ date fruit at the kimri stage was observed because of the pollen from ‘Jarvis’ and ‘Shahani’. At the khalal stage, the highest content was observed in fruits created by the pollen from ‘Jarvis’. At the rutab and tamar stages, the highest content was observed in fruits created by the pollen from ‘Jarvis’ and ‘kabkab’ cultivars (Table 4). The lowest amount of chlorophyll a in the kimri stage was obtained with pollen of ‘Beraem’ and ‘Faryab’, in the khalal stage with pollen of ‘Fard’, ‘Sheikhali’ and ‘Zahedi’ and, in the rutab and tamar stages, with the pollen of ‘Fard’ cultivar.

**Table 4:** Chlorophyll a content of ‘Piarom’ date fruit in kimri, khalal, rutab and tamar stages after pollination with eight pollinating cultivars.

Cultivars	Kimri (mg/100 g)	Khalal (mg/100 g)	Rutab (mg/100 g)	Tamar (mg/100 g)
‘Shahani’	1.45 a	0.75 d	0.27 cd	0.25 cd
‘Kabkab’	1.27 c	0.87 ab	0.37 a	0.35 a
‘Zahedi’	1.37 b	0.69 e	0.26 cde	0.23 de
‘Beraem’	1.04 d	0.85 bc	0.35 ab	0.33 ab
‘Faryab’	1.07 d	0.82 c	0.31 bc	0.28 c
‘Sheikhali’	1.35 b	0.68 e	0.23 de	0.21 de
‘Fard’	1.23 c	0.67 e	0.21 e	0.20 e
‘Jarvis’	1.47 a	0.90 a	0.37 a	0.35 a

Different letters within columns indicate significant differences according to Duncan’s multiple range test (p < 0.05).

The highest amount of chlorophyll b in ‘Piarom’ date fruit was obtained at the kimri stage with pollen from ‘Jarvis’ and ‘Shahani’, at the khalal stage with pollen from ‘Jarvis’, at the rutab stage with pollen from ‘Jarvis’, ‘Kabkab’ and ‘Beraem’. At the tamar stage, the highest was obtained in fruits created by pollen from ‘Jarvis’ and ‘kabkab’ cultivars. The lowest amount of chlorophyll b in ‘Piarom’ date fruits was obtained at the kimri stage with pollen from ‘Beraem’ and ‘Faryab’, and at the khalal, rutab and tamar stages with pollen from ‘Fard’ and ‘Sheikhali’ cultivars (Table 5).

**Table 5:** Chlorophyll b content of ‘Piarom’ date fruit in kimri, khalal, rutab and tamar stages after pollination with eight pollinating cultivars.

Cultivars	Kimri (mg/100 g)	Khalal (mg/100 g)	Rutab (mg/100 g)	Tamar (mg/100 g)
‘Shahani’	0.34 a	0.30 ab	0.25 ab	0.23 ab
‘Kabkab’	0.32 ab	0.32 ab	0.27 a	0.25 a
‘Zahedi’	0.33 ab	0.30 ab	0.25 ab	0.23 ab
‘Beraem’	0.31 b	0.31 ab	0.27 a	0.24 ab
‘Faryab’	0.31 b	0.31 ab	0.26 ab	0.24 ab
‘Sheikhali’	0.33 ab	0.28 b	0.23 b	0.21 b
‘Fard’	0.32 ab	0.28 b	0.23 b	0.21 b
‘Jarvis’	0.34 a	0.33 a	0.28 a	0.25 a

Different letters within columns indicate significant differences according to Duncan’s multiple range test (p < 0.05).

#### Fruit carotenoid

The highest amount of carotenoid in ‘Piarom’ date fruits, after pollination with the pollen of the eight cultivars, was observed at the kimri stage with pollen of ‘Fard’ and ‘Sheikhali’. In the khalal stage, it was with pollen from ‘Fard’, in the rutab stage with pollen from ‘Fard’ and ‘Sheikhali’ and, in the tamar stage, with pollen from ‘Fard’ cultivar (Table 6). Also, the lowest amount of carotenoids in ‘Piarom’ date fruits was obtained at the kimri stage with pollen from ‘Kabkab’, Beraem’ and ‘Faryab’. At the khalal stage, it was with pollen from ‘Kabkab’, ‘Beraem’, ‘Faryab’ and ‘Jarvis’. At the rutab stage, with pollen of ‘Kabkab’ and at the tamar stage with pollen of ‘Kabkab’ and ‘Beraem’ cultivars. In general, the amount of carotenoids in ‘Piarom’ date fruits increased after pollination, from the kimri to the khalal stage, but decreased in the rutab and tamar stages. The highest amount of carotenoids, regardless of pollen source, was observed at the khalal stage, which is the coloring stage of date fruit growth (Table 6).

**Table 6:** The amount of carotenoids of 'Piarom' date fruit in kimri, khalal, rutab and tamar stages after pollination of eight pollinating cultivars.

Cultivars	Kimri (mg/100 g)	Khalal (mg/100 g)	Rutab (mg/100 g)	Tamar (mg/100 g)
‘Shahani’	0.41 ab	0.73 bc	0.11 bcd	0.10 bc
‘Kabkab’	0.39 b	0.71 c	0.09 d	0.07 d
‘Zahedi’	0.41 ab	0.73 bc	0.13 ab	0.10 bc
‘Beraem’	0.39 b	0.71 c	0.10 cd	0.08 d
‘Faryab’	0.40 b	0.72 c	0.11 bcd	0.09 cd
‘Sheikhali’	0.42 a	0.75 ab	0.14 a	0.11 ab
‘Fard’	0.42 a	0.76 a	0.15 a	0.12 a
‘Jarvis’	0.41 ab	0.72 c	0.11 bcd	0.09 cd

Different letters within columns indicate significant differences according to Duncan’s multiple range test (p < 0.05).

#### Fruit anthocyanin

The highest amount of anthocyanin in date fruits of the ‘Piarom’ date cultivar, after pollination with the pollen of the eight pollinating cultivars, was obtained at the kimri, khalal, rutab, and tamar stages with pollen of ‘Shahani’, ‘Sheikhali’, ‘Zahedi’, ‘Fard’, ‘Jarvis’ and ‘Faryab’, respectively (Table 7).

**Table 7:** The amount of anthocyanin in ‘Piarom’ date fruit in kimri, khalal, rutab and tamar stages after pollination with eight pollinating cultivars.

Cultivars	Kimri (mg/100 g)	Khalal (mg/100 g)	Rutab (mg/100 g)	Tamar (mg/100 g)
‘Shahani’	0.081 a	0.084 a	0.066 a	0.037 a
‘Kabkab’	0.061 b	0.062 b	0.045 b	0.022 b
‘Zahedi’	0.080 a	0.084 a	0.065 a	0.037 a
‘Beraem’	0.063 b	0.065 b	0.045 b	0.022 b
‘Faryab’	0.075 a	0.083 a	0.063 a	0.036 a
‘Sheikhali’	A0.08 1	0.084 a	0.067 a	0.037 a
‘Fard’	0.080 a	0.083 a	0.064 a	0.036 a
‘Jarvis’	0.080 a	0.083 a	0.064 a	0.036 a

Different letters within columns indicate significant differences according to Duncan’s multiple range test (p < 0.05).

The lowest amount of anthocyanin was obtained at the kimri, khalal, rutab, and tamar stages with pollen from the ‘kabkab’ and ‘Beraem’ cultivars.

#### Fruit reducing and non-reducing sugars

The highest amount of glucose in ‘Piarom’ date fruit at the tamar stage was observed after pollination with pollen of ‘Sheikhali’, ‘Fard’ and ‘Zahedi’ and the lowest amount was observed with pollen of ‘Kabkab’ and ‘Beraem’ cultivars, which showed a significant difference with other cultivars at the probability level of 5% (Table 8). The highest amount of fructose sugar in ‘Piarom’ date fruits was produced at the tamar stage after pollination with pollen of ‘Sheikhali’ and ‘Fard’ cultivars, which was not significantly different from the amount of fruit fructose with pollen of ‘Zahedi’ cultivar and the lowest amount was observed with pollen of ‘Kabkab’ and ‘Beraem’ cultivars, which showed a significant difference at the level of 5% probability, compared to the other cultivars (Table 8). The highest amount of sucrose sugar in ‘Piarom’ date fruits at the tamar stage was observed after pollination with pollen of ‘Jarvis’ cultivar and the lowest amount was observed in fruits created by pollen from ‘Kabkab’ cultivar, which showed a significant difference at the probability level of 5%, compared to other cultivars (Table 8).

**Table 8:** The amount of glucose, fructose and sucrose sugars in ‘Piarom’ date fruit (tamar stage) after pollination with eight pollinating cultivars.

Cultivars	Glucose (g/100 g)	Fructose (g/100 g)	Sucrose (g/100 g)
‘Shahani’	32.75 c	22.67 b	0.79 d
‘Kabkab’	29.09 e	20.07 d	0.57 e
‘Zahedi’	35.02 a	23.13a b	1.23 c
‘Beraem’	29.44 e	20.39 d	0.65 de
‘Faryab’	30.35 d	21.47 c	0.72 d
‘Sheikhali’	35.47 a	23.57 a	1.61 b
‘Fard’	35.28 a	23.38 a	1.27 c
‘Jarvis’	34.24 b	22.71 b	1.90 a

Different letters within columns indicate significant differences according to Duncan’s multiple range test (p < 0.05).

#### Fruit polygalacturonase enzyme

The highest activity of polygalacturonase enzyme in ‘Piarom’ date fruits, after pollination with the pollen of the eight pollinating cultivars, at the kimri stage, was observed with pollen of ‘Fard’, ‘Sheikhali’, ‘Zahedi’ and ‘Shahani’. At the khalal, rutab and tamar stages, it was with the pollen of ‘Fard’ cultivar which showed a significant difference compared to the other cultivars at the probability level of 5% (Table 9). In addition, the lowest amount was created at the kimri, khalal, rutab, and tamar stages, with pollen from ‘Kabkab’ and ‘Beraem’ cultivars, respectively, which showed a significant difference at the level of 5% probability, compared to the other cultivars.

**Table 9:** Polygalacturonase enzyme activity of ‘Piarom’ date fruit in kimri, khalal, rutab and tamar stages after pollination with eight pollinating cultivars.

Cultivars	Kimri (IU/g.FW)	Khalal (IU/g.FW)	Rutab (IU/g.FW)	Tamar (IU/g.FW)
‘Shahani’	2.39 a	2.48 b	6.49 c	7.10 c
‘Kabkab’	2 d	2.08 d	3.84 f	4.04 f
‘Zahedi’	2.40 a	2.49 b	6.98 b	7.21 c
‘Beraem’	2.02 d	2.09 d	3.87 f	4.09 f
‘Faryab’	2.10 c	2.21 c	4.67 e	5.20 e
‘Sheikhali’	2.43 a	2.49 b	7.05 b	8.09 b
‘Fard’	2.45 a	2.54 a	8.20 a	8.77 a
‘Jarvis’	2.20 b	2.27 c	5.92 d	6.26 d

Different letters within columns indicate significant differences according to Duncan’s multiple range test (p < 0.05).

#### Fruit cellulase enzyme

The highest activity of cellulase enzyme in ‘Piarom’ date fruits, after pollination with the pollen of eight pollinating cultivars, at the kimri stage, was obtained with pollen from ‘Fard’ and ‘Zahedi’, at the khalal and rutab stages with pollen from ‘Fard’ and at the tamar stage with pollen from ‘Fard’ and ‘Zahedi’ cultivars (Table 10). The lowest activity of cellulase enzyme was obtained at the kimri, khalal, rutab, and tamar stages, with pollen from the ‘Beraem’ cultivar.

**Table 10:** Cellulase enzyme activity of ‘Piarom’ date fruit in kimri, khalal, rutab and tamar stages after pollination with eight pollinating cultivars.

Cultivars	Kimri (IU/g FW × 10^–3^)	Khalal (IU/g FW × 10^–3^)	Rutab (IU/g FW × 10^–3^)	Tamar (IU/g FW × 10^–3^)
‘Shahani’	209.75 ab	497.75 d	521 d	582 bc
‘Kabkab’	195.75 ab	424.50 e	435.75 fg	495.25 e
‘Zahedi’	220.25 a	660 b	682.75 b	830.75 a
‘Beraem’	181.75 b	380.50 f	407.50 g	440.75 f
‘Faryab’	204 ab	433.50 e	452.50 ef	518.50 de
‘Sheikhali’	213.25 ab	549.75 c	569 c	607 b
‘Fard’	228.50 a	778 a	791.50 a	866 a
‘Jarvis’	206 ab	470 d	489.75 de	554.75 cd

Different letters within columns indicate significant differences according to Duncan’s multiple range test (p < 0.05).

#### Fruit invertase enzyme

The highest invertase enzyme activity in ‘Piarom’ date fruits, after pollination with the pollen of the eight pollinating cultivars, at the kimri stage, was observed with pollen from ‘Sheikhali’ and ‘Fard’, at the khalal stage, with pollen from ‘Sheikhali’, at the rutab stage, with pollen from ‘Sheikhali’ and ‘Fard’ and, at the tamar stage, with pollen from ‘Sheikhali’ which did not show a significant difference compared to the amount of this enzyme in fruits created by the pollen of ‘Fard’ cultivar (at the level of 5% probability) (Table 11). At the kimri stage, the lowest amount was observed in fruits created by pollen from ‘Kabkab’. At the khalal stage, it was with pollen from ‘Kabkab’ and ‘Beraem’, at the rutab stage with pollen from ‘Kabkab’ and in the tamar stage with pollen from ‘Kabkab’, ‘Beraem’ and ‘Faryab’ cultivars which had the highest amount of invertase enzyme at the different stages of kimri, khalal, and tamar, compared to the other cultivars, and showed a significant difference at the level of 5% probability.

**Table 11:** Invertase enzyme activity of ‘Piarom’ date fruit in kimri, khalal, rutab and tamar stages after pollination with eight pollinating cultivars.

Cultivars	Kimri (IU/g.FW)	Khalal (IU/g.FW)	Rutab (IU/g.FW)	Tamar (IU/g.FW)
‘Shahani’	0.60 d	0.92 e	0.83 c	0.68 d
‘Kabkab’	0.28 f	0.37 g	0.27 e	0.26 e
‘Zahedi’	1.16 b	1.55 c	1.17 b	1.16 b
‘Beraem’	0.31 ef	0.44 g	0.33 de	0.28 e
‘Faryab’	0.42 e	0.72 f	0.41 d	0.36 e
‘Sheikhali’	1036 a	1.80 a	1.60 a	1.35 a
‘Fard’	1028 a	1.67 b	1.49 a	1.28 ab
‘Jarvis’	0.84 c	1.06 d	0.94 c	0.83 c

Different letters within columns indicate significant differences according to Duncan’s multiple range test (p < 0.05).

## Discussion

### Pollen grains characteristics

According to the results, the pollen of different cultivars had different amounts of carbohydrates. This can be due to differences in genetic structures and growth conditions, especially the age and growth potential of the plants that produced them. The most important carbohydrates in pollen are sucrose, glucose, and fructose. Simple sugars are used as a source of energy to help pollen germinate. Carbohydrates are important compounds for the growth and development of pollen. Impaired sugar metabolism in anthers can lead to harmful effects on pollen and eventually causes sterility^33^. In this study, the pollen of cultivars that contained higher amounts of carbohydrates also had desirable and better conditions for the fertilization of female organs in ‘Piarom’ date trees and, subsequently, this led to the production of high-quality fruits.

### Starch is a glucose polymer

In the anther of the flower stamen, starch is considered as the most important sugar storage for microsporangium. The decomposition of starch usually takes place according to the type of plant species; the time of decomposition is usually during microsporogenesis and after or regularly during the growth and development of pollen^34^. Therefore, decomposition of starch in pollen grains can provide the necessary sugars for pollen germination and fertilization processes^35^. In our study, differences in the amount of starch in the pollen of different pollinator cultivars may be due to differences in genetic structure and plant growth conditions. On the other hand, due to the fact that starch reserves in pollen are considered as a source of energy for pollen germination and pollen tube growth, cultivars such as ‘Fard’ and ‘Shahani’, which had large amounts of starch, caused better fertilization and fertility in ‘Piarom’ trees.

Protein and starch in pollen are considered as primary sources of energy for pollen grain germination and pollen tube growth^36^. Small pollen grains usually contain more protein, and large pollen grains have more starch, both of which are effective in pollen tube growth^37^. In species which pollen is high in protein, the pollen tube growth can occur rapidly and, conversely, in species which pollen is low in protein, there is usually a slow rate of pollen tube growth^38^. Proteins and lipids in the pollen exine can affect the rate of pollen tube growth in egg cells^37^. In this study, the presence of different amounts of proteins in the pollen could also be due to genetic conditions along with different growth and developmental conditions of plants that produce them. Pollen in some cultivars contain higher amounts of protein due to the role of proteins in the process of pollen adhesion to the surface of stigma cells. These pollen tend to have more effective interactions with the pistil flowers of ‘Piarom’ date trees after pollination.

Pollen and date fruits have antioxidant properties due to their phenolic compounds, flavonoids, carotenoids and anthocyanin^39^. In plants, these compounds protect plant cells against oxidative stress caused by free radicals^40^. Phenolic compounds are one of the most important compounds that exhibit antioxidant activity in pollen. Flavonoids are also the largest group of phenolic compounds in nature and are found in free or glycoside forms^41^. Flavonoids are plant secondary metabolites of polyphenols derived from the phenylpropanoid pathway and are a group of antioxidants^42^. Flavonoids are produced in the pollen of tapetum tissue. The biosynthesis of these compounds is essential for the normal growth of pollen. The mutant plants, which were unable to synthesize flavonoids, produced sterile male plants with white pollen. In addition, pollen without flavonoids does not grow well^43^. In this study, the difference in the amount of phenolic substances and flavonoids in the pollen of different cultivars of date palm pollinators was due to differences in genetic structure, as well as the growth and development of plants that produced them. Pollen of cultivars that contain higher amounts of these compounds have desirable quality characteristics for fertilization and fertility of ‘Piarom’ date trees.

The cell wall at the end of the pollen tube is mainly composed of methyl-esterified pectins which are responsible for the high flexibility of the pollen tube tip, and for enabling the possibility of expanding and developing it^44^. The pectin methyl esterase enzyme is a protein derived from the endoplasmic reticulum, which plays a major role in causing significant changes in the cell wall of the pollen tube. This enzyme also plays a major role in the formation of cell walls in plants^45^.

Pollen are considered as physiological systems. The growth and germination of pollen on the stigma of female organs are affected by environmental conditions such as temperature, water content, UV rays, and food reserves. Nutrient reserves in pollen are used during pollen tube growth and germination. Therefore, enzymes must be present to digest the nutrient stores in the pollen as well as to decompose the surrounding pectin membrane^46^. The amylase enzyme breaks down starch particles into glucose for consumption and absorption. Starch is a major source of carbon and a major source of carbohydrates^47^. In this study, differences in pectin methylesterase and alpha-amylase enzymes were observed in the pollen of different date pollinator cultivars. This was probably caused by differences in the genetic origin and growth conditions of the plants that produce them. The pollen of cultivars that contain large amounts of pectin methyl esterase enzyme can promote effective longitudinal growth of the pollen tubes. In addition, the pollen of cultivars that contain higher amounts of alpha-amylase can effectively break down the starch reserves in pollen to facilitate proper longitudinal growth of the pollen tube. Therefore, the use of such pollen during pollination on ‘Piarom’ palm trees can lead to effective fertilization and fertility, followed by the production of high-quality fruits.

### Fruit characteristics

In this study, pollination with pollen from all eight pollinating cultivars produced the highest amount of phenolic compounds at the kimri stage and, gradually, with progress toward the khalal, rutab, and tamar stages, the amount of phenolic compounds decreased, reaching the lowest level at the tamar stage. A decrease in the amount of phenolic compounds, parallel to the progress in fruit growth and development, were similarly observed among date cultivars in the United States, Saudi Arabia^48^ and Tunisia. In this regard, Amira et al.^11^ showed that the khalal stage had the highest amount of phenolic compounds compared to the rutab and tamar stages. In a study on several date cultivars, it was observed that the amount of phenolic compounds in fruit flesh was reduced by 25% from the khalal to the tamar stage^49^. According to Al-Turki et al.^48^, the amount of phenolic compounds in date fruits varied between different cultivars and different stages of fruit development. The reduction of phenolic compounds in date fruits from the khalal to the tamar stage was reportedly observed because of the oxidation of these compounds by polyphenol oxidase enzyme^50^, as well as the decrease in soluble tannins^51^.

In our study, pollen grains of ‘Jarvis’ and ‘Shahani’ cultivars, which led to the production of higher amounts of phenolic compounds in the fruit, showed desirable characteristics and were compatible with ‘Piarom’ date trees. This led to the production of fruits with favorable metabolic conditions. The presence of small amounts of phenolic compounds in the fruits of ‘Piarom’ dates, which were caused by the pollen of ‘Kabkab’ and ‘Beraem’ cultivars, can be due to lower quality characteristics and less compatibility with pollen grains of these cultivars.

Changes in tannin compounds during the development of date fruits play a decisive role in the usability of date fruits during the khalal or later stages of fruit development^49^. According to previous studies^52,53^, the use of pollen from different pollinators during the date pollination process leads to the production of different amounts of tannins at different stages of fruit development. However, the amount of fruit tannin under pollination with different pollinators decreased from the kimri to the khalal, rutab, and tamar stages. Tannins can form reversible bonds with polysaccharides, proteins, and alkaloids, owing to their hydroxyl groups^49^. The balance between soluble and insoluble tannins plays an important role in determining the edibility of date fruit, because high concentrations of tannins may make dates less edible^54^. In our study, it was observed that the use of pollen from different date pollinator cultivars led to changes in fruit tannin levels during the different stages of growth and development of ‘Piarom’ date fruits. After pollination by pollen from all eight pollinating cultivars, the amount of tannin in the fruit decreased from the kimri to the tamar stage. Also, pollen of ‘Fard’ and ‘Sheikhali’ cultivars led to the production of the lowest amounts of fruit tannin in most stages of growth and development of ‘Piarom’ date fruits. In contrast, ‘Kabkab’ and ‘Beraem’ cultivars produced the highest amount of fruit tannin in most of these stages. This condition can be due to favorable quality conditions and, furthermore, because the pollen of ‘Fard’ and ‘Sheikhali’ cultivars were more compatible with ‘Piarom’ date trees. This can also be explained by the production of fruits with better growth and metabolic conditions, which may be mainly due to the production of higher amounts of hormones in them^55^. Finally, with the pollen of ‘Fard’ and ‘Sheikhali’ cultivars, fruits with the least amount of tannins are produced, which is a desirable biochemical feature.

Pollination of ‘Piarom’ palm trees with pollen of eight pollinating cultivars resulted in different amounts of chlorophyll a and b at the different stages of fruit development. The highest amount of chlorophyll a and b of ‘Piarom’ date fruit was generally observed at the kimri stage, after pollination, regardless of the pollinator cultivar. The lowest amount, however, was observed at the tamar stage. This could be due to the enzymatic degradation of chlorophyll during the ripening stage of the fruit. It was also observed that pollen of ‘Jarvis’ and ‘Kabkab’ cultivars, which led to higher chlorophyll production in ‘Piarom’ date fruits, also had late-ripening fruits. On the other hand, pollen of ‘Fard’ and ‘Sheikhali’ cultivars, which resulted in lower chlorophyll content in ‘Piarom’ date fruits, produced early-ripening fruits. Therefore, it seems that the pollen of ‘Fard’ and ‘Sheikhali’ cultivars produced optimal amounts of chlorophyllase enzyme and were able to degrade chlorophyll in a shorter period of time than other cultivars. Early in the development of the fruits, it is known that the chlorophyllase enzyme binds to the inner membrane of the chloroplast and, thus, loses access to the chlorophylls in the thylakoids. During the maturation and aging process of a fruit or leaf, chloroplast continuity is lost and the chlorophyllase enzyme comes into direct contact with chlorophyll, which eventually causes its decomposition^56^. In a study on ‘Zaghloul’ date cultivar^57^, it was reported that the use of pollen of different cultivars during pollination leads to changes in the amount of chlorophyll a and b of the fruit during different stages of growth and development. In addition, in fruits produced by all pollinators, a significant decrease in chlorophyll a and b was observed from the kimri stage to the khalal. On the other hand, non-pollinated fruits had higher levels of chlorophyll a and b at different stages of fruit development compared to pollinated fruits. These results were consistent with those of the present study.

Carotenoids such as anthocyanins, polyphenols and flavonoids are low molecular weight antioxidant compounds that have an effective role in neutralizing free radicals, leading to the protection of intracellular compounds against the damaging effects of free radicals^14^. In a relevant research, it was reported that the amount of carotenoids decreased rapidly in ripe dates, and anthocyanins were found only in fresh dates^58^. In addition, through the growth and development of plant tissues towards maturation and aging, compounds such as pigments, proteins, and phospholipids usually decrease, which may be due to their decomposition by free radicals; at this stage of development, the plant protection system is reduced to neutralize free radicals^59^. According to a relevant study, the amount of carotenoids was reportedly high during the early growth and development of date fruits, but then decreased during fruit ripening^60^. Under environmental stresses that lead to the production of different types of reactive oxygen species in plant cell chloroplasts, the presence of carotenoids protects photosynthetic tissues and chlorophylls^61,62^. In the present study, pollen of ‘Fard’ and ‘Sheikhali’ cultivars led to the production of the highest amount of carotenoids in ‘Piarom’ date fruits. It seems that the pollen of these cultivars have favorable morphological and physiological characteristics and, also, they are more compatible with ‘Piarom’ palm trees. Thus, they led to the production of fruits with desirable quantitative and qualitative characteristics such as the presence of increased amounts of carotenoids. On the other hand, the presence of the highest amount of carotenoids at the khalal stage can be due to the major decomposition of chlorophyll at this stage, which leads to the appearance of carotenoids in the fruit. Carotenoids were produced at the early stages of date palm growth at the kimri stage but, due to the presence of large amounts of chlorophyll a and b, they did not have the opportunity to appear. In other words, carotenoids were hidden in the chlorophyll coating.

The amount of anthocyanin in ‘Piarom’ date fruits after pollination with the pollen of the eight pollinating cultivars increased from the kimri stage to the khalal. This increase was such that it peaked at the khalal stage, but decreased thereafter to the rutab and tamar stages. Also, different levels of anthocyanin were produced in ‘Piarom’ date fruits after pollination with pollen of the different pollinating cultivars, which shows the effect of pollen grain metaxenia on the characteristics of ‘Piarom’ date fruit. Since there are sugar units in the molecular structure of anthocyanin, the production of these pigments depends on the presence of sunlight and the amount of carbohydrates in the plant^63^. In our study, pollen, which led to the synthesis of more sugars in ‘Piarom’ fruit, also produced higher levels of anthocyanin in the fruit. In a study on the characteristics of ‘Zaghloul’ date fruit under the influence of different pollinators, it was reported that pollen from different pollinators led to changes in the amount of anthocyanin in date fruits during the different stages of fruit development. In this study, the anthocyanin content increased with the developmental stage of the date fruit. In addition, the pollen of these pollinators produced the highest amount of fruit anthocyanin at the khalal stage^57^, which is consistent with the results of the current study.

In the present study, using pollen from different cultivars led to the production of different amounts of glucose, fructose and sucrose in ‘Piarom’ date fruits. This trend may indicate the effect of pollen grain metaxenia on the activity of invertase enzyme that breaks down sucrose into glucose and fructose. The pollen of ‘Sheikhali’, ‘Fard’ and ‘Zahedi’ cultivars in comparison with the pollen of ‘Kabkab’ and ‘Beraem’ led to the highest levels of glucose and fructose in ‘Piarom’ date fruits. Carbohydrates in date fruits mainly contain reducing sugars such as glucose and maltose, as well as non-reducing sugars such as sucrose, and small amounts of polysaccharides such as cellulose and starch^39^. The amount of reducing sugars in date fruits is considered an important criterion for determining their quality. The most abundant types of reducing sugars in most date cultivars are glucose and fructose^64^. In all date cultivars, the amount of sugars increases during fruit development and reaches its maximum at the end of fruit maturity^65^. One study reported that date fruit sugar can be affected by different pollinator sources during pollination. In ‘Hayany’ cultivar, for example, the highest amount of total sugar and reduced sugar contents were obtained in fruits that resulted from the pollen of ‘M1’ cultivar^66^. In addition, date fruits that resulted from the pollen of different pollinating cultivars had more reduced sugars, as well as more non-reduced and total sugars, compared to non-pollinated fruits^53^.

In general, the activity of polygalacturonase and cellulase enzymes in date fruits increases from the initial stages of fruit development to the stages of maturity and fruit ripening. The process of softening in fruit tissues during the ripening stage, by cell wall expanding proteins, increases the activity of enzymes such as polygalacturonase and cellulase in the cell wall. Different date cultivars have very low levels of polygalacturonase in the green stage of the fruit. The major increase in the activity of this enzyme occurs when the date fruit changes from green to yellow, or to other colors, depending on the cultivar (at the khalal stage). Therefore, an increase occurs in the activity of this enzyme in date fruits, especially in the final stages of fruit ripening, leading to a rapid decrease in fruit firmness^67^. In this study, using pollen from different pollinator cultivars led to the production of different amounts of polygalacturonase activity at different stages of fruit growth and development. This could be due to the metaxenia effect of pollen grains of different cultivars on the activity of the polygalacturonase enzyme. Pollen of the ‘Fard’ cultivar produced the highest amount of polygalacturonase activity in most stages of fruit growth and development. Therefore, it seems that the pollen of ‘Fard’ cultivar had more favorable physiological conditions and more compatibility with ‘Piarom’ palm trees, compared to ‘Kabkab’ and ‘Beraem’ cultivars. The pollen of ‘Fard’ cultivar led to the production of fruits with high levels of polygalacturonase enzyme activity, which was essentially needed to decompose pectin compounds and soften fruit tissues during maturity and fruit ripening.

The enzyme cellulase is commonly present in the fruits of all plant species, and together with the enzyme polygalacturonase, it plays an important role in the process of softening fruit tissues during the ripening stage^68^. According to a previous study on ‘Lonet Mesaed’ dates, cellulase enzyme activity was at a relatively low level at the early stages of date fruit development, but then its activity increased significantly and reached its maximum at the khalal stage, thereafter declining slightly at the rutab stage^69^. In our study, using pollen from different date pollinator cultivars led to the production of different amounts of cellulase enzyme activity at different stages of growth and development of the ‘Piarom’ dates. This trend can be attributed to the metaxenia effect of the different pollen on cellulase enzyme activity. The pollen of ‘Fard’ and ‘Zahedi’ cultivars led to the highest levels of cellulase activity in ‘Piarom’ date fruits, compared with the pollen of ‘Beraem’ cultivar. This observation indicated that these pollinators are more compatible with ‘Piarom’ trees.

The softening of fruit tissues during the fruit ripening process, in addition to the amount of moisture, also depends on the activity of a number of hydrolyzing enzymes such as invertase^70^. Invertase enzymes convert sucrose to reducing sugars (glucose and fructose). The highest amount of invertase enzyme was observed during the ripening stage of date fruits (at the khalal stage). Invertase activity is higher in soft date cultivars than in semi-dry dates^67^. Invertase enzyme activity in the ‘Hillawi’ cultivar was reportedly not active at the early stages of date fruit development. However, the highest level of activity was observed at the fruit ripening stage (khalal), and the lowest level of activity was observed at the tamar stage^71^. In general, the highest invertase enzyme activity was observed during the khalal stage, but decreased during the rutab and tamar stages^67^. In a study on ‘Shahani’ and ‘Piarom’ cultivars, it was reported that the amount of invertase enzyme increased sharply from the kimri to the khalal stage and then decreased during the rutab and tamar stages^72^. In the present study, using pollen from different pollinator cultivars led to different amounts of invertase enzyme activity during the stages of fruit growth and development. This condition can be due to the metaxenia effect of pollen grains of different cultivars on the level of invertase activity in ‘Piarom’ date fruits. In this study, the activity of invertase enzyme in ‘Piarom’ date fruit increased generally, regardless of pollen source. It increased from the kimri to the khalal stage, but then decreased at the rutab and tamar stages. Also, the pollen of ‘Sheikhali’ and ‘Fard’ cultivars, compared with the pollen of ‘Kabkab’ and ‘Beraem’ cultivars, led to the highest amount of invertase activity at different stages of fruit growth, thereby showing desirable quality characteristics and greater compatibility with ‘Piarom’ date trees.

## Conclusion

Different pollinator cultivars of date palm create pollen with different constituents and characteristics due to their genetic origin, diverse growth patterns and variable developmental conditions. In this study, the eight pollinators differed in carbohydrates, starch, proteins, total phenols, flavonoids, and enzymes such as pectin methylesterase and amylase. The pollen of ‘Sheikhali’, ‘Fard’, ‘Zahedi’ and ‘Shahani’ had more of the above substances than the pollen of other cultivars. The effects of pollen from these four cultivars were observable on the characteristics of fruits, as the pollen fertilized ‘Piarom’ date flowers. The fruits that formed as a result of fertilization by these pollen were superior to fruits obtained from pollen of other cultivars in terms of secondary metabolites, sugars and enzymes. In particular, the pollen of ‘Fard’ and ‘Sheikhali’ produced the best fruits in terms of secondary metabolites. These results confirm the metaxenia effects of date pollen grains on fruit formation in the same year.
